# Effects of Varicocelectomy on Serum Testosterone Levels among Infertile Men with Varicocele 

**DOI:** 10.22074/ijfs.2018.5058

**Published:** 2018-03-18

**Authors:** Meysam Jangkhah, Faramarz Farrahi, Mohammad Ali Sadighi Gilani, Seyed Jalil Hosseini, Farid Dadkhah, Reza Salmanyazdi, Mohammad Chehrazi

**Affiliations:** 1 Department of Embryology, Reproductive Biomedicine Research Center, Royan Institute for Reproductive Biomedicine, ACECR, Tehran, Iran; 2 Department of Andrology, Reproductive Biomedicine Research Center, Royan Institute for Reproductive Biomedicine, ACECR, Tehran, Iran; 3 Department of Urology, Shariati Hospital, Tehran University of Medical Sciences, Tehran, Iran; 4 Infertility and Reproductive Health Research Center, Shahid Beheshti Medical University, Tehran, Iran; 5 Department of Epidemiology and Reproductive Health, Reproductive Epidemiology Research Center, Royan Institute for Reproduc- tive Biomedicine, ACECR, Tehran, Iran

**Keywords:** Infertility, Testosterone, Varicocele, Varicocelectomy

## Abstract

**Background:**

The main purpose of this study is to evaluate the effects of varicocelectomy on serum testoster- one levels and semen quality in infertile men who suffer from varicocele.

**Materials and Methods:**

This prospective study enrolled 115 subjects with clinical varicocele grades II and III and 240 fertile men as the control group. Total volume of testosterone serum level (ng/dl) and semen quality were com- pared before and after microscopic varicocelectomy. We normalized testosterone serum levels for age, grade, and testis size basis. SPSS 20 software was used to analyze the data. All results of continuous variables were reported as mean ± SD. Statistical significance was set at a P<0.05.

**Results:**

The mean ages of individuals who participated in the treatment (32.2 ± 5.23) and control (32.8 ± 5.27) groups were similar. There were similar mean values for adjusted testosterone levels between the varicocele (567 ± 222 ng/ml) and control (583 ± 263 ng/ml) groups. In the varicocele group, the adjusted testosterone levels insig- nificantly increased to 594 ± 243 ng/ml. Among semen parameters, only mean sperm concentration significantly increased after varicocelectomy.

**Conclusion:**

Despite increases in sperm concentration, adjusted testosterone levels did not significantly improve after varicocelectomy.

## Introduction

The relationship between varicocele and male infertility was first noted in the late 1800s when Bennet reported an improvement in semen quality after correction for bilateralvaricoceles in a patient (1, 2). Varicocele is an abnormaldilatation of the pampiniform plexus of the veins that drain the testis. Restoration of this abnormality has been shown to cause positive effects on the spermatogenesis process (2-4). According to a number of studies, varicocelectomy improves semen parameters, hormonal profiles, and pregnan. cy rates (5-8). However, the process by which varicocele and its repair affects testicular Leydig cell function, semen quality, and the resultant changes in testosterone produc. tion levels are less understood and intensely debated. Many studies have reported that varicocelectomy promotes Leydig cell function based on testosterone levels. In addition, research indicates that ageing in men can induce a reduction in serum testosterone levels (2, 9, 10).

Among mechanisms involved in controlling testicular testosterone level, temperature has been highlighted. Animal models showed that both varicocele and increased testicular temperatures impede sperm production (5, 11). Disruption in the cooling system in veins of the scrotum during varicocele results in an increase in temperature of the scrotum. This phenomenon can be overcome by varicocelectomy (5, 8, 11). High temperatures can reduce the activity of the 17-a hydroxyl progesterone aldolase en. zyme, which results in decreased testosterone production. Thus, it is believed that treatment of varicocele may improve the function of Leydig cells, reactivate this enzyme, and increase testosterone production (12-14). In light of this understanding, we aim to assess the effects of varicocelectomy on serum testosterone levels and semen quality in infertile men with varicocele.

## Materials and Methods

### Patients and group design

We conducted this prospective research on 115 infertile men with clinical varicocele grades II and III and 240 fertile men as the control group. The study received approval from the Ethical Committee (number: EC/91/1114) of Royan Institute (Tehran, Iran) and was conducted from August, 2012 to February, 2015. The subjects were men, ages 21-46 years, who were not affected by diabetes and did not take medications known to elicit imbalanced androgen levels. The control group included men who had one or more children, did not suffer from varicocele and diabetes, and did not take medications known to elicit changes in androgen levels. Prior to performing the study, consent letters were received from the patients which informed them of all the study procedures. We included another control group, called the witness group, as the positive control that compared testosterone hormone levels between non-varicocele treated fertile men (had at least one child in the recent year or had more children during their coupling life) against infertile men diagnosed with varicocele.

### Blood sample collection and testosterone assay

Patients and fertile males provided blood samples and we compared their serum testosterone levels. The blood samples of infertile men were taken 3-6 months after surgery in order to reassess the changes in serum testosterone levels. Semen parameters (concentration, motility, and morphology) were assessed according to WHO guidelines. In infertile men, prior to varicocelectomy, we assessed the effects of age, testis size (left-right), and grade on the mean total testosterone level. Semen samples were obtained by masturbation after 3-5 days of sexual abstinence. Accordingly, the patient’s samples were taken before and after varicocelectomy to evaluate the effects of varicocele repair upon the quality of the sperm parameters.

Blood samples were taken from fertile and infertile men. The level of total testosterone was evaluated by an Elisa Kit (AccuBind® Microwell ELISA Kit, Monobind Inc., Lake Forest, CA, USA) before and after (3-6) varicocelectomy. The sample group (individuals with varicocele) was categorized into two groups according to testis volume of the patients with the volume < 16 ml (2). We also characterized the study group members into two groups based on age less than 35 years old and more than 35 years old. 

### Statistical analysis

The Pearson correlation was applied to specify the relationship between continuous variables, and the independent t test was used to compare testosterone levels, age, and semen parameters between infertile men with varicocele and fertile men. The unit of testosterone is ng/dl. SPSS 16 software was used to analyze the data. The paired t test was performed to compare the pre- and post-operative testosterone levels, semen volumes, sperm concentrations, and motility. All results of the continuous variables were reported as mean standard deviation. Statistical significance was set at a P<0.05. Multiple linear regression analysis was applied to identify potential factors that affected the changes in mean testosterone levels before surgery.

## Results

**Fig.1:**
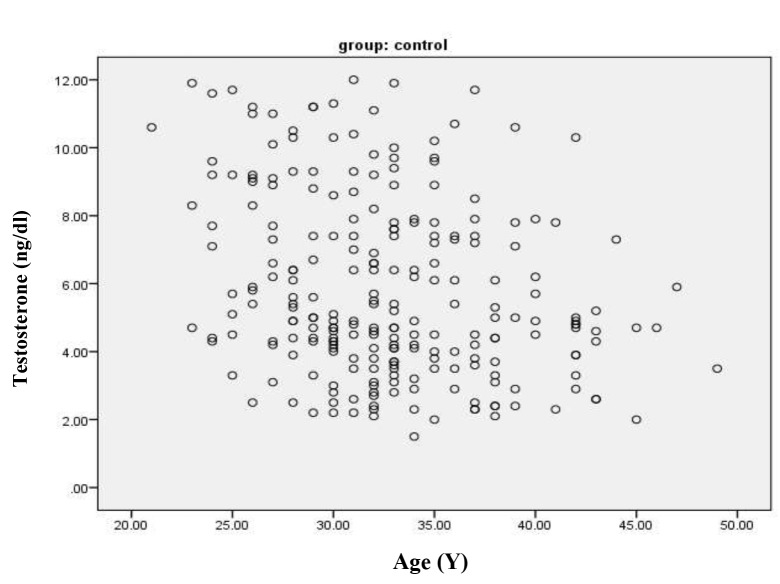
Scatter plot that demonstrates the relationship between testosterone and age in the control group (P<0.05).

**Fig.2:**
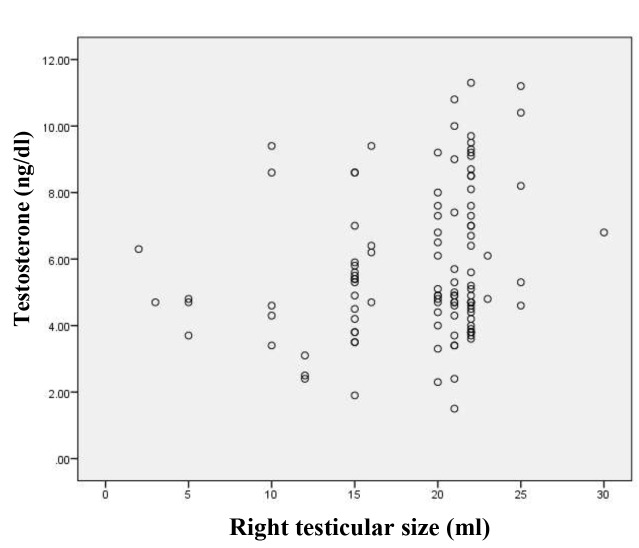
Scatter plot that demonstrates the relationship between testosterone and right testis size in infertile men with varicocele before surgery (P<0.05).

A total of 355 men participated in the study-240 control and 115 infertile men with varicocele. Fertile men had higher mean testosterone levels (583 ± 263 ng/dl) compared to infertile men (567 ± 222 ng/dl) before the operation, however this was not a statistically significant difference (P=0.558). The mean ages of infertile (32.2 ± 5.23 years) and fertile men (32.8 ± 5.27 years) were not significantly different (P=0.328). There was a significant linear relationship observed between age and testosterone level among the control group (Fig .1, r=-0.28, P<0.0001), but we did not observe this in the varicocele group (r=- 0.17, P=0.07). The mean size of the left testes (18.58 ± 4.98) was statistically lower than the right testes (19.01 ± 4.75, P=0.017). Pearson correlation showed a significant correlation between total testosterone and right testis size (r=0.21, P=0.026) in infertile men with varicocele before surgery (Fig .2). There was no relationship between grade of varicocele and testosterone level (r=-0.05, P=0.58). Varicocelectomy resulted in an insignificant rise in testosterone levels from 567 ± 222 ng/dl to 594 ± 243 ng/dl (P=0.27, Table 1). 

**Table 1:** Comparison of testosterone and semen parameters before and after varicocelectomys


Variable	Before surgery Mean ± SD	After surgery Mean ± SD	P value

Testosterone (ng/dl)	567 ± 223	594 ± 243	0.27
Volume	3.29 ± 1.67	3.39 ± 1.80	0.47
Sperm concentration (×10^6^/ml)	19.10 ± 23.50	28.90 ± 31.90	0.00
Sperm motility (%)	31.60 ± 24.60	32.30 ± 25.60	0.66


Semen parameters that included including: volume, motility, and concentration were assessed before and after surgery. Both volume and motility of the sperm nonsignificantly increased after surgery. However, sperm concentration significantly (P<0.001) increased after surgery. Linear regression was used to show the effects of the variables (age, grade, and testis size) on total testosterone before surgery (Table 2). The other model was selected using the backward method (Table 3). The regression coefficient for age and right testis size compared to testosterone as the dependent variable was significant (P=0.036). 

**Table 2:** Correlation between testosterone concentrations before surgery with age, grade of varicocele, and left and right testis sizes


Variable	Coefficient	P value

Age	-0.166	0.077
Left testis size	0.177	0.063
Right testis size	0.211	0.026
Varicocele grade	-0.052	0.579


**Table 3:** Multivariable linear regression coefficients for testosterone before surgerys


Variable	Coefficient	Standard error	P value

Age	-0.067	0.039	0.092
Right testis size	0.087	0.041	0.036


## Discussion

The relationship between varicocele and disorder in the function of testosterone production was not clearly understood in that work. To the best of our knowledge, few or no studies have assessed the effect of varicocelectomy upon Leydig cell function and testosterone production. Treatment of varicocele may lead to a suitable condition on total testosterone levels (2). As shown in our research, despite the increased testosterone level in infertile men after varicocelectomy, the difference was not significant. 

Other researchers reported the negative impact of varicocele on spermatogenesis. In order to improve the quality of sperm parameters, varicocelectomy was used to treat male infertility. Therefore, we evaluated the other parameters that supposedly affect total testosterone levels. These parameters included age, grade, and testis size. We determined that the difference in the sizes of the left and right testes impacted total testosterone level in infertile men. According to previous studies, the probability of varicocele increased with increased age (15-17). Hsiao et al. (18) showed that the testosterone levels lower than 400 ng/dl improved considerably in individuals after varicocele treatment. However, it has been shown in earlier works that varicocelectomy may improve testosterone production even if it is not significant in addition to semen quality, particularly sperm concentration and motility (8, 9, 19). The present research has shown that the preoperative testosterone levels in infertile men were lower compared to fertile men. After surgery, testosterone levels increased in infertile men with varicocele. However, this increase was not significant. Other sperm parameters such as volume, motility and concentration were analyzed pre- postoperative. Although all parameters increased, only the increase in sperm concentration was statistically significant.

In addition to statistical analysis of the mentioned components, we assessed multivariable linear regression coefficients for testosterone before surgery by taking into consideration age and right testis size. Although the coefficient regression related to age stood negative, it was not significant. There was a significant relation between right testis size and total serum testosterone level.

Resorlu et al. (20) recently reported no changes in serum testosterone levels after varicocele repair. Notably, low-normal testosterone values were recorded both before and after the repair with no significant change in serum testosterone levels for any of their study groups. Preoperative and 6-month postoperative subjects were evaluated by Rodriguez Peña et al. (21), in which hormonal profiles and other data showed an increase in the serum testosterone after surgery, but this increase was not significant. Zohdy et al. (22) reported that patients who underwent varicocelectomy demonstrated a significant postoperative improvement in serum testosterone levels. Considering that varicocele has been universally accepted to negatively impact testis function, including paracrine and endocrine functions of the Leydig cells, the relationship between varicocele and diminished androgen levels appears to be reversed with varicocele repair. However, further studies are needed to better understand the multifactorial pathophysiology of varicocele-mediated Leydig cell dysfunction (23).

## Conclusion

The results of this study show that varicocelectomy could improve sperm parameters such as sperm concentration and increase the testosterone level of blood serum although the increase is statistically insignificant. Nevertheless, it appears that this treatment is necessary to improve function in testes afflicted with varicocele.
